# Kenya tuberculosis prevalence survey 2016: Challenges and opportunities of ending TB in Kenya

**DOI:** 10.1371/journal.pone.0209098

**Published:** 2018-12-26

**Authors:** Masini Enos, Joseph Sitienei, Jane Ong’ang’o, Brenda Mungai, Maureen Kamene, Jesse Wambugu, Hillary Kipruto, Veronica Manduku, Josephine Mburu, Drusilla Nyaboke, Faith Ngari, Eunice Omesa, Newton Omale, Nkirote Mwirigi, Geoffrey Okallo, Janice Njoroge, Martin Githiomi, Mike Mwangi, Dickson Kirathe, Richard Kiplimo, Amos Ndombi, Lazarus Odeny, Eunice Mailu, Timothy Kandie, Maurice Maina, Kadondi Kasera, Beatrice Mulama, Beatrice Mugi, Herman Weyenga

**Affiliations:** 1 Ministry of Health, Nairobi, Republic of Kenya; 2 Kenya Medical Research Institute, Nairobi, Kenya; 3 Center for Health Solutions, Kenya; 4 Africa Hologic, Nairobi, Kenya; 5 World Health Organization Kenya country office, Nairobi, Kenya; 6 United States Agency for International Development, Nairobi, Kenya; 7 Kenyatta National Hospital, Nairobi, Kenya; 8 US Centers of Disease Control and Prevention, Nairobi, Kenya; Universidad Nacional de la Plata, ARGENTINA

## Abstract

**Background:**

We aimed to determine the prevalence of pulmonary TB amongst the adult population (≥15 years) in 2016 in Kenya.

**Method:**

A nationwide cross-sectional survey where participants first underwent TB symptom screening and chest x-ray. Subsequently, participants who reported cough >2weeks and/or had a chest x-ray suggestive of TB, submitted sputum specimen for laboratory examination by smear microscopy, culture and Xpert MTB/RIF.

**Result:**

The survey identified 305 prevalent TB cases translating to a prevalence of 558 [95%CI 455–662] per 100,000 adult population. The highest disease burden was reported among people aged 25–34 years (716 [95% CI 526–906]), males (809 [(95% CI 656–962]) and those who live in urban areas (760 [95% CI 539–981]). Compared to the reported TB notification rate for Kenya in 2016, the prevalence to notification ratio was 2.5:1. The gap between the survey prevalence and notification rates was highest among males, age groups 25–34, and the older age group of 65 years and above. Only 48% of the of the survey prevalent cases reported cough >2weeks. In addition, only 59% of the identified cases had the four cardinal symptoms for TB (cough ≥2 weeks, fever, night sweat and weight loss. However, 88.2% had an abnormal chest x-ray suggestive of TB. The use of Xpert MTB/RIF identified 77.7% of the cases compared to smear microscopy’s 46%. Twenty-one percent of the survey participants with respiratory symptoms reported to have sought prior health care at private clinics and chemists. Among the survey prevalent cases who reported TB related symptoms, 64.9% had not sought any health care prior to the survey.

**Conclusion:**

This survey established that TB prevalence in Kenya is higher than had been estimated, and about half of the those who fall ill with the disease each year are missed.

## Introduction

Tuberculosis (TB) remains a global threat to public health and is the leading cause of death by a single infectious agent with 1.6 million deaths in 2017. An estimated 10 million people developed TB disease in 2017 but only 6.4 million (61%) were notified [1]. The global TB targets aim at a 95% reduction in TB deaths, 90% reduction in incidence compared to 2015 and 0% TB affected families facing catastrophic costs due to TB by 2035 [2]. To achieve these, the true burden of TB needs to be ascertained so that efforts to find all incident cases are scaled up. In countries without high quality vital registration and health notification systems, TB prevalence surveys offer the best method to accurately measure the TB burden.

Kenya is listed by the World Health Organization (WHO) among the 30 high burden TB states [3]. Despite the considerable investment done by the government and partners in TB care and prevention in the past 20 years, the disease is still the 4th leading cause of death [4]. Finding all people with TB disease and successfully treating them is therefore an important priority for the country.

Kenya conducted the last national TB prevalence survey in 1958 and has thus relied on estimates from WHO to extrapolate TB incidence and case detection rates. At that time, the prevalence of TB was 3,142 per 100.000 population (110.000 cases in a population of 3.5 million aged 10 years or more) [5]. In 2015, the country’s pre-TB prevalence survey incidence of TB was 233 per 100 000 (95% CI 188–266) while the country was estimated to detect 80% of all TB cases [3].The drivers of TB have certainly changed over the past 60 years. Recent TB surveys had been conducted in HIV prevalent areas and had limited geographic scope making generalization of their findings to the whole country difficult. Nonetheless, their results suggested that TB incidence in Kenya was being underestimated [6].

The pre-survey estimates of the TB burden in Kenya were based on modeling that used routine notification data and a number of assumptions including known or estimated annual risk of TB infection, HIV prevalence and socio-economic factors. Considering the known limitations of routine TB data, these estimates were unreliable and were of limited use for country specific planning. In 2012, Kenya was one of the 22 high priority countries selected by the WHO to undertake a national TB prevalence survey [7]. The survey was eventually undertaken in 2016.

The specific objectives of the nationwide tuberculosis prevalence survey were to determine the prevalence of bacteriologically confirmed pulmonary TB (PTB) among adults aged 15 years and above in Kenya and to assess the health care seeking behavior of TB patients and those reporting TB symptoms.

This survey provides more accurate TB prevalence estimates as well as insights on the associated health seeking behavior of TB patients and those reporting symptoms. The survey further characterizes persons identified with TB that are not yet detected by the National TB Program (NTP) while providing a platform for measuring the impact of TB control activities and progress towards meeting TB control targets. The findings provide a rare opportunity to critically re-engineer TB control strategies and provide a robust response towards the detection and treatment of all TB cases, placing Kenya on the road towards ending TB.

## Materials and methods

The Kenya TB prevalence survey was a nationwide cluster-based, cross-sectional study conducted between July 2015 and July 2016 with a sample size of 72,000 individuals aged 15 years and above.

### Target population

The target population comprised of all persons (males and females) aged 15 years and above residing in Kenya drawn from the selected clusters. Household members who had lived in the household for a minimum of 30 consecutive days prior to the date of the survey and who consented were recruited into the survey. People institutions requiring special clearance (prisons, police/military/National Youth Service camps, health facilities, diplomatic compounds, schools, refugee camps, hotels and lodgings) were excluded.

### Sample size population

Calculation of the sample size was based on the following assumptions: prevalence of smear positive TB in adults (≥ 15 years) of 268.7 per 100,000 [8], a relative precision to be tolerated at 95 per cent level of confidence of 20%, a design effect of 1.7 and an expected 85% participation rate. One hundred clusters were sampled by probability proportional to size method with the households in each cluster being the measure of size. Sampling of the clusters was done independently in the urban and rural strata. Rural areas were geographic areas located outside towns and cities. Agricultural areas were considered rural. Urban areas referred to cities, towns and surrounding their suburbs. Ultimately 32 (30%) urban clusters and rural 68 (70%) clusters were selected, reflecting their general population share.

### Survey field procedures

In each cluster, the field work started with mapping of the cluster boundaries. A door-to-door census using the listing questionnaire was then conducted in all the households within the cluster enumerating all eligible persons aged 15 years until the target number of 650–790 adults per cluster was reached. The listing questionnaire was used to capture details of all members of the households in the selected clusters including the characteristics of each person listed, such as age and sex. A social economic questionnaire was then administered to the household head. The socio-economic questionnaires were used to collect household information which would assist during the calculation of wealth quintiles. All eligible consenting household members were thereafter invited to a nearby mobile field site (MFS).

### Mobile field site procedures

Individuals who were eligible to participate in the survey underwent the WHO recommended screening strategies for TB prevalence surveys: symptom questionnaire and chest X-ray at the MFS [9]. The symptom screening and health seeking behaviour questionnaires were administered to all survey participants. The symptom screening assessed presence of cough, cough duration, sputum production, heamoptysis, night sweats, chest pain, fever, fatigue, difficulty in breathing and weight loss. Upon enrolment, participants were assigned unique study identification numbers that were used throughout the mobile field site processes.

Thereafter all survey participants consenting to chest radiography had a digital chest X-ray done with opt out approach for pregnant women. Chest X-ray images were independently evaluated using a standardized WHO criteria, by two trained clinical officers as either normal, abnormal suggestive of TB or abnormal other.

Those who were symptomatic (i.e. cough for 2 or more weeks), and/or had abnormal chest X-ray suggestive of TB, and those who declined or could not undergo chest X-ray were requested to submit sputum specimens for laboratory examination. All survey cases were referred to the nearest health facility for TB treatment including HIV counselling and testing as per the Kenyan TB guidelines.

### Laboratory procedures

Two sputum samples (spot and morning from the next day) were obtained. Both specimens were transported under cold chain to National Tuberculosis Reference Laboratory (NTRL) in Nairobi for smear microscopy, Xpert MTB/RIF and solid culture using Lowenstein Jensen (L-J) medium. A direct smear was done on all samples and stained with auramine O then followed by microscopic examination at (20x and 40x magnification) using a fluorescent microscope. Xpert MTB/RIF was done on all morning samples and on spot samples lacking a matching morning sample. Both the spot and morning sputum specimens underwent solid culture examination. The samples for culture were first decontaminated using 4% sodium hydroxide and then processed. Each sample was inoculated on two slopes of L-J medium, incubated at a temperature of 37°C and monitored weekly for growth for a period of up to eight weeks. A culture was only reported negative if there was no growth after eight weeks. Except for samples that were contaminated, all visible colonies grown on culture media were confirmed by acid-fast bacilli (AFB) microscopy and Mycobacterium protein 64 (MPT64) speciation to confirm presence of MTB complex. Subsequent susceptibility testing on the Mycobacterium Growth Indicator Tube (MGIT) was performed to rule out resistance to first line drugs (streptomycin, isoniazid, rifampicin and ethambutol). Non-Tuberculosis Mycobacterium (NTM) and preliminary resistance to rifampicin and isoniazid were identified using Geno-Type Mycobacterium AS and GenoType MTBDRplus (Hain Life science) test kits.

### Quality assurance/control

All radiographers and clinical officers underwent training by the consultant radiologists for standardization of the radiological procedures and evaluation of the chest radiographs. In addition, regular supervision of the field teams was also done to ensure quality.

All chest X-rays that were abnormal, 10% of normal and all images with discordant findings between the 2 field clinical officers were read by 2 central consultant radiologists. In situations of disagreement between the 2 radiologists, a third radiologist did the final reading. All the images of bacteriologically confirmed TB cases were re-read and their clinical information discussed by the case management team.

### Data collection and management

All the questionnaires were pre-loaded in the field computers that facilitated digital data collection. A unique identification number called study identification number (Study ID) was used in all the stages of data collection and data management. The ID was converted into a barcode and used on all forms/register and in the digital data files to identify each survey participant. The survey generated mostly digital data with the exception of participant invitation cards and informed consent forms.

Data from both household listing and MFS were electronically transmitted first to the MFS and from the MFS to the central data management unit (DMU). Digital X ray images were assigned specific system generated numbers that were linked to the study ID numbers. These images were electronically transmitted to the central server. These images were later transmitted to the DMU and processed for central reading.

The DMU also received data electronically from the NTRL.

The central data management team monitored the survey database to ensure routine and timely data collection. They also participated in field supervision and liaised with the field data managers to ensure a seamless data collection process.

The data collected was stored in secure server at the National TB Program with authorized access only. Data cleaning was performed continuously using SAS code. Any data discrepancies were discussed and resolved by the data monitoring unit in consultation with the field team.

### Data analysis

Data was analysed using STATA version 14. Descriptive statistics on eligibility, enrollment and demographics was performed. The outcomes of screening and sputum testing were described. Core analysis focused on estimating the prevalence of pulmonary TB in the adult population. The 3-model approach as recommended by the WHO was applied (9). In this article only model 3 (random-effect logistic regression) results are presented. This model used robust standard errors with missing value imputations and inverse probability weighting. Where inverse probability weighting corrected for differentials in participation in the survey by age, sex, and cluster. This model took account of both clustering and variation in the number of individuals per cluster, when estimating both the point prevalence of pulmonary TB and its 95% confidence interval.

### Ethical considerations

The study protocol was approved by the scientific and ethical review committee of the Kenya Medical Research Institute (KEMRI). The approval reference was SSC 2094. Administrative approval from the various county health committees was obtained. Informed consent was obtained from each household head before administration of the socioeconomic questionnaire. Written informed consent was obtained from each eligible participant before enrolment into the study and after thorough explanation of the risks and benefits of participating in the study. Any questions raised by the potential participants were answered before participation in the study. The voluntary nature of participation in the survey and the option to withdraw from the study at any time without affecting participant rights and benefits was explained. Consent for eligible participants with low or no literacy was obtained in the presence of a witness and signatures obtained using their thumb print. Consent for minors aged 15 to 17 years was sought from a parent or guardian and individual assent obtained. Mature minors (married, pregnant, parent, head of a household) provided individual consent as specifically approved by the ethics committee. Radiation safety procedures were applied including protection and monitoring of the workers and participants. All TB cases and participants needing care were referred to the nearest health facility.

## Results

**Fig 1:**
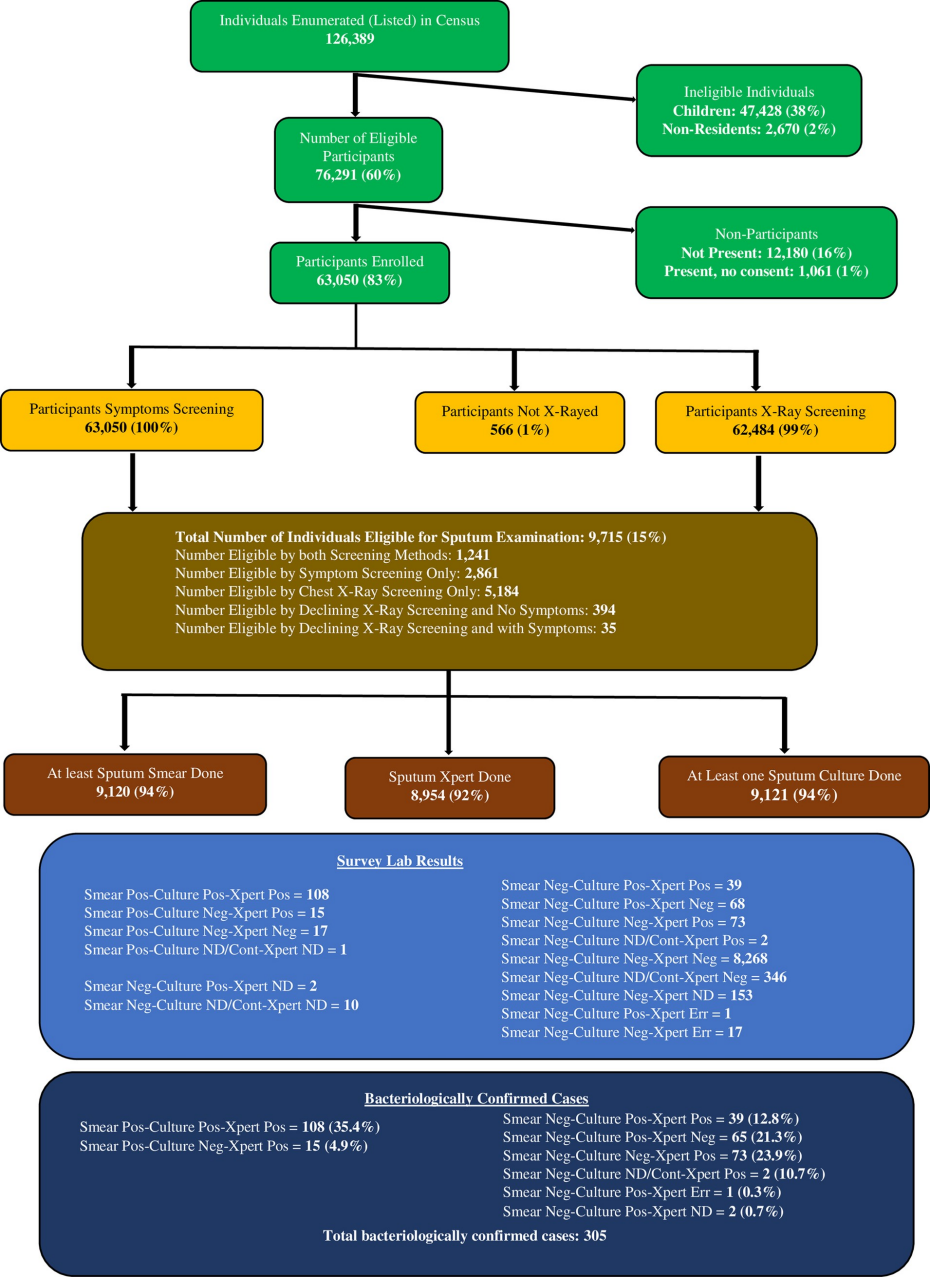
Schematic diagram of number of participants screened for TB in the survey.

In total, 126,389 individuals were registered in the survey census. Of these 76,291 (60%) were eligible to participate in the survey, of whom 63,050 (83%) participated. Females comprised of 59% of the participants with a higher participation rate of 87% compared to 77% in males. All the 63,050 participants underwent symptom screening, of which chest X-ray was conducted on 62,484 (99%) of the participants. In total of 9,715 (15%) participants were eligible for sputum submission of whom 9,120 (94%) submitted at least one sputum specimen, while 7,763 (80%) were able to submit both (Fig 1).

Of the enrolled participants, 24,256 (38%) reported at least one symptom, 9,305 (15%) reported coughing and 7% had a cough for more than 2 weeks. The most frequently reported symptoms were; chest pain 19%, cough 15%, drenching night sweats 12%, fatigue 11%, fever 8%, cough with sputum 5% as shown in Table 1.

**Table 1:** Frequency of TB related symptoms among survey participants.

Symptoms	Number	% of enrolled
Cough > 2 weeks	4,137	7
Chest pain	12,290	19
Coughing	9,305	15
Drenching night sweats	7,357	12
Fatigue	7,228	11
Cough < 2weeks	5,168	8
Fever	4,937	8
Cough with sputum	3,256	5
Shortness of Breath	3,417	5
Weight loss	1,609	3
Other symptoms	1,114	2
Hemoptysis (Blood Cough)	393	1
**Total symptomatic**	**24,256**	38
**Total enrolled**	**63,050**	

Among the sputum eligible survey participants n = 9,715, 74% (7,185) reported at least one symptom. The most common symptom was cough of 2 weeks or more (43%), followed by chest pain (41%), cough with sputum (32%), drenching night sweats (27%), fatigue (27%), fever (19%), shortness of breath (11%), weight loss (8%) and hemoptysis (4%). Of the sputum eligible, 5,184 (53%) were eligible through chest X-ray findings only, 2,896 (30%) were through symptoms only, 1,241 (13%) by both X-ray and symptoms while 394 (4%) were eligible because they declined X-ray screening though asymptomatic.

Smear microscopy and culture was done for 9,120 (94%) persons whilst 8,954 (92%) had Xpert MTB/RIF test as shown in Table 2. Xpert MTB/RIF had the highest number of positive results (2.7%) followed by culture with 2.4% and microscopy at 1.6% (Table 2). Contamination rate was 3.9%.

**Table 2:** Laboratory examination results.

Laboratory method	Results	Freq. (%)	Spot (%)	Morning (%)
**Smear**	POS	141 (1.6)	140 (1.6)	131 (1.7)
	NEG	8,979 (98.5)	8,834 (98.4)	7,777 (98.3)
	**Total**	**9,120**	**8,974**	**7,908**
**Xpert MTB/RIF**	MTB	237 (2.7)	235 (3.0)	218 (2.5)
	MTB Not Detected	8,699 (97.2)	7,623 (96.8)	8,557 (97.3)
	Error	9 (0.1)	16 (0.2)	17 (0.2)
	Invalid	8 (0.1)	0	0
	Not Done	1 (0.1)	0	0
	**Total**	**8,954**	**7,874**	**8,792**
**Culture**	MTB	218 (2.4)	216 (2.4)	197 (2.5)
	NTM	236 (2.6)	0	0
	No Growth reported	8,307 (91.1)	8,427 (93.9)	7,462 (94.4)
	Contaminated	359 (3.9)	331 (3.7)	249 (3.1)
	**Total**	**9,120**	**8,974**	**7,908**

The survey identified a total of 305 bacteriologically confirmed pulmonary TB prevalent cases resulting in a weighted TB prevalence rate of 558 [455–662] per 100,000 adult population. Males had more than twice the prevalence rate compared to females; 809 per 100,000 adult population against 359 per 100,000 adult population (Table 3).

**Table 3:** Overview of weighted pulmonary TB prevalence adults aged 15 years and above per 100,000 population in Kenya.

		Smear positive only (n = 123); Rate per 100,000 (95% CI)	Xpert MTB/RIF only (n = 237) Rate per 100,000 (95% CI	Bacteriological Confirmed (N = 305) Rate per 100,000 (95% CI)
1	**National**	230 (174,286)	431 (353,509)	558 (455,662)
2	**Sex**			
	Male	346 (260,431)	614 (498,729)	809 (656,962)
	Female	138 (79,196)	286 (202,370)	359 (258,460)
3	**Age**			
	15–24	218 (133,303)	311 (206,416)	360 (242,478)
	25–34	259 (164,353)	530 (381,679)	716 (526,906)
	35–44	297 (164,430)	484 (319,649)	602 (422,782)
	45–54	234 (101,367)	492 (327,656)	607 (432,781)
	55–64	118 (24,211)	313 (159,467)	587 (372,803)
	65+	125 (24,226)	449 (264,634)	576 (368,783)
4	**Setting**			
	Urban	335 (213,456)	603 (439,767)	760 (539,981)
	Rural	175 (126,224)	341 (268,414)	453 (357,549)

**Fig 2:**
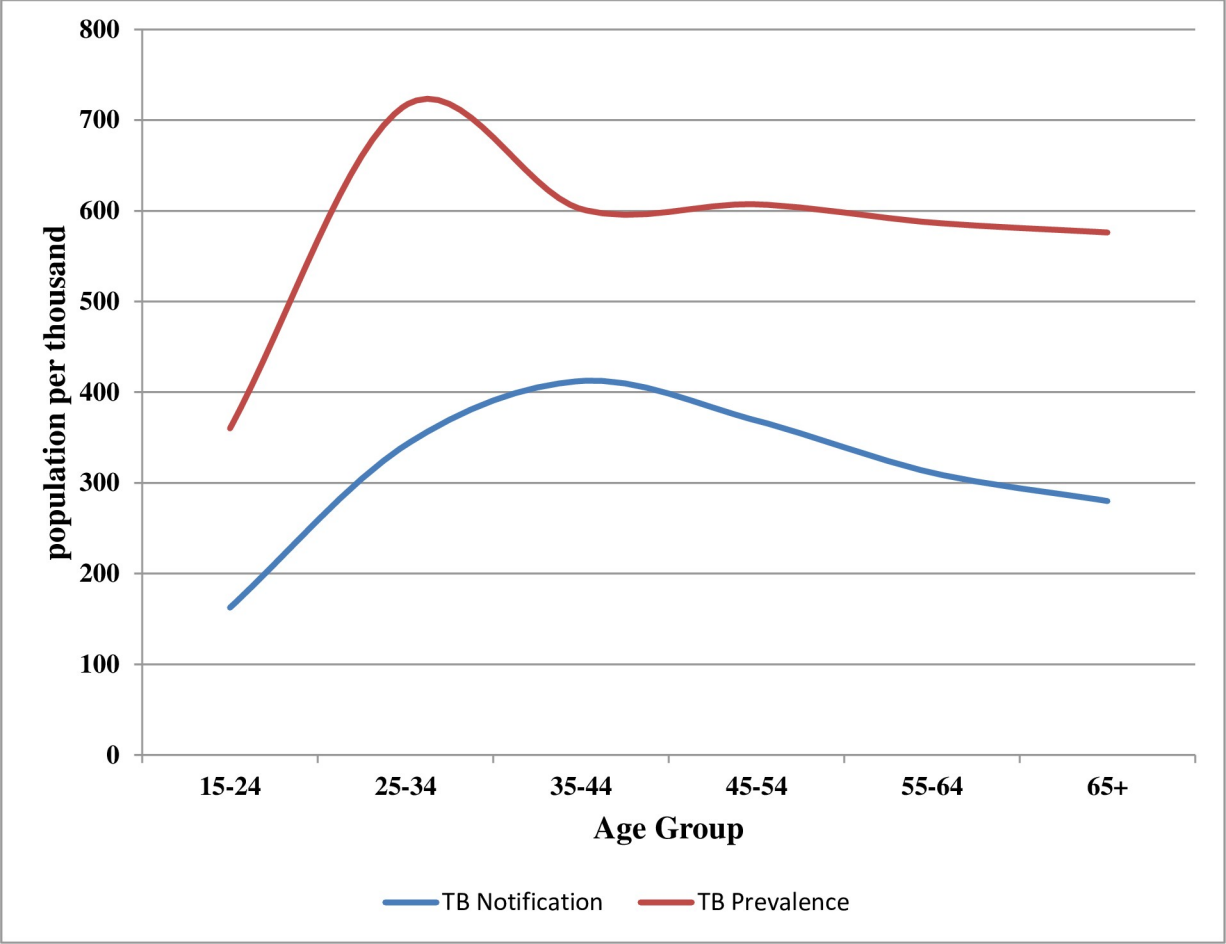
Distribution of TB prevalence and case notification by age group.

The age group 25–34 years had the highest prevalence rate of 716 [526–906] per 100,000 adult population compared to the other age groups. Comparison of the distribution of the survey TB prevalence rate and the Kenya case notifications rates for 2015 by age group showed that notification rates were lower than the survey prevalence rates. The gap between prevalence and notification rates was highest among age groups 25–34 and the older age group of 65+ years (Fig 2).

Forty eight percent of the survey prevalent cases (n = 147) reported cough of lasting more than 2 weeks while 28 (9%) reported a cough of less than 2 weeks (Table 4). Forty-three percent of the prevalent cases reported no history of cough. Digital chest X-ray was able to detect 269 (88.2%) and missed 29 (9.5%) of the prevalent cases while 7 (2.3%) had no chest X-ray done (Table 4). New cases contributed the highest number of prevalent cases at 219 (72%) while previously treated cases accounted for 71 (24%). Five percent (n = 15) of the identified TB cases were on current TB treatment.

**Table 4:** Proportions of prevalent cases by screening methods (symptoms and chest x-ray).

	Bacteriologically confirmed Cases	Percentage of prevalent cases
**Symptoms Present**		
Cough > = 2 Weeks	147	**48.2**
Cough < 2 Weeks	28	**9.2**
No Cough	130	**42.6**
**Overall**	**305**	**100**
**Field X-ray Results**		
Abnormal suggestive of TB	269	**88.2**
Normal	24	**7.9**
Abnormal others	5	**1.6**
Declined/Missing	7	**2.3**
**Overall**	**305**	**100**

If cough of ≥2weeks were to be used as the sole screening method, 158 (52%) of the survey prevalent cases would have been missed (Table 5). Use of a combination of the four TB cardinal symptoms (cough ≥2 weeks, fever, night sweat and weight loss) only for screening, 124 (41%) of the cases would have been missed. Use of any TB related symptom for screening would have missed only 80 (26%) of the prevalent cases.

**Table 5:** Symptoms profile of the prevalent TB cases.

Symptom	Cases	%
Cough > two weeks only	147	48
Night sweats only	85	28
Fever only	62	20
Weight loss only	41	13
Weight loss or fever or night sweats or cough more than two weeks	181	59
Any coughing or fever or weight loss or night sweats or fatigue or other symptoms or breathe shortness or chest pains (At least one symptom)	225	74
**Total**	**305**	**100**

People with TB were 4.5 times more likely to be symptomatic than people without TB at both unadjusted and adjusted analyses (adjusted 95% CI: 3.46, 5.81]) (Table 6). Before adjustments, all TB symptoms listed except cough for less than 2 weeks, seemed to be associated with being diagnosed with TB. However, after adjusting for demographics and co-symptoms, being diagnosed with TB remained significantly associated with the following: cough of any duration (adjusted OR: 3.75, 95% [CI: 2.74, 5.25]), cough with sputum (adjusted OR: 2.72, 95%[CI: 1.95, 3.79]), and weight loss (adjusted OR: 2.36, 95% [CI: 1.62, 3.45]).

**Table 6:** Association between being diagnosed with tuberculosis with presence of tuberculosis-related symptoms.

Symptoms	N of cases	Un-Adjusted OR	Un-Adjusted 95%CI	p-value	Adjusted OR	Adjusted 95%CI	p-value
Coughing	175	7.91	(6.29, 9.93)	< 0.0001	3.75	(2.74, 5.15)	< .0001
Cough > 2 weeks	147	13.70	(10.92, 17.19)	< 0.0001			
Cough < 2weeks	28	1.13	(0.77, 1.67)	0.5302			
Cough with sputum	112	11.00	(8.69, 13.92)	< 0.0001	2.72	(1.95, 3.79)	< .0001
Hemoptysis(Blood Cough)	18	10.43	(6.41, 16.98)	< 0.0001	1.06	(0.62, 1.8)	0.8452
Weight loss	41	6.06	(4.34, 8.45)	< 0.0001	2.36	(1.62, 3.45)	< .0001
Drenching night sweats	85	2.95	(2.29, 3.79)	< 0.0001	1.18	(0.87, 1.6)	0.2999
Fatigue	77	2.63	(2.03, 3.4)	< 0.0001	1.05	(0.77, 1.44)	0.7672
Fever	62	3.03	(2.29, 4.01)	< 0.0001	1.22	(0.87, 1.7)	0.2431
Shortness of Breath	45	3.05	(2.22, 4.19)	< 0.0001	1.29	(0.91, 1.82)	0.1473
Chest pain	128	3.01	(2.39, 3.78)	< 0.0001	1.11	(0.85, 1.47)	0.4439
Other symptoms	12	2.29	(1.28, 4.09)	0.0040	1.13	(0.61, 2.07)	0.7056
Total symptomatic	225	4.53	(3.51, 5.85)	< 0.0001	4.48	(3.46, 5.81)	< .0001

Tables 7 and 8 describe the concordance and level of agreement between Xpert MTB/RIF and culture among the 305 TB cases identified by the survey. Forty-eight percent of these cases were detected by both methods. A total of 90 cases (29.5%) were diagnosed by Xpert MTB/RIF but were missed by culture while 65 (21.3%) were diagnosed by culture but missed by Xpert MTB/RIF. There was substantial agreement between Xpert MTB/RIF and culture in TB diagnosis (Cohen’s kappa statistic, k = 0.64, 95% CI = 0.59, 0.70) (Table 7)

**Table 7:** Comparison of TB yield by diagnostic methods among the 305 prevalent cases.

**Prevalent cases**	** **		**Culture results**			
			**Culture MTB Positive**	**Culture Negative**	**Contaminated**	**Grand Total**
	**Xpert MTB/RIF results**	**MTB Positive**	147 (48.2%)	88 (28.9%)	2 (0.8%)	**237 (77.7%)**
		**MTB Negative**	65 (21.3%)	-	-	**65 (21.3%)**
		**Error**	1 (0.3%)	-	-	**1 (0.3%)**
		**Not Done**	2 (0.7%)	-	-	**2 (0.7%)**
		**Grand Total**	**215 (70.5%)**	**88 (28.9%)**	**2 (0.7%)**	**305 (100%)**
**Smear positive cases**	** **		**Culture results**			
			**Culture MTB Positive**	**Culture Negative**	**Contaminated**	**Grand Total**
	**Xpert MTB/RIF results**	**MTB Positive**	111 (76.0%)	15 (10.3%)	-	**126 (86.3%)**
		**MTB Negative**	-	18 (12.3%)	-	**18 (12.3%)**
		**Error**	-	-	-	-
		**Not Done**	-	1 (0.7%)	1 (0.7%)	**2 (1.4%)**
		**Grand Total**	**111 (76.0%)**	**34 (11.1%)**	**1 (0.7%)**	**146 (100%)**
**Smear negative cases**	** **		**Culture results**			
			**Culture MTB Positive**	**Culture Negative**	**Contaminated**	**Grand Total**
	**Xpert MTB/RIF results**	**MTB Positive**	39 (0.4%)	73 (0.8%)	2 (0.0%)	**114 (1.3%)**
		**MTB Negative**	68 (0.8%)	8,268 (96.7%)	346 (3.9%)	**8,682 (96.7%)**
		**Error**	1 (0.0%)	17 (0.2%)	0	**18 (0.2%)**
		**Not Done**	2 (0.0%)	153 (1.7%)	10 (0.1%)	**165 (1.8%)**
		**Grand Total**	**110 (0.1)**	**8,511 (94.8%)**	**358 (4.0%)**	**8,979 (100%)**

**Table 8:** Tthe level of agreement between Xpert MTB/RIF and culture in TB diagnosis.

		Culture				
		Positive	Negative	Total		
**Xpert MTB/RIF**	Positive	147	90	237	Cohen’s Kappa	0.64
	Negative	68	8,649	8,739	95%CI	(0.59, 0.70)
	Total	215	8,739	8,954		

After adjusting for settings, sex, age groups, occupation, level of education, and marital status, people with TB diagnosed through smear microscopy were significantly more likely to be symptomatic, as compared to Xpert MTB/RIF and culture (adjusted ORs of 2.73 [p<0.003], 1.13 [p = 0.4] and 1.41 [p = 0.08] respectively) (Table 9). Those diagnosed through smear microscopy were significantly more likely to have cough of any duration and weight loss (adjusted OR of 2.56 and 3.21 respectively, p<0.05). People with TB as diagnosed by culture showed similar trend (adjusted OR of 2.13 and 2.14 respectively, p<0.05). Conversely, people with TB as diagnosed by Xpert MTB/RIF were more likely to have weight loss and shortness of breath (adjusted OR of 1.89 and 1.58 respectively, p<0.05).

**Table 9:** Association between the method of TB diagnosis and presence of TB related symptoms.

	Number of cases			Un-adjusted OR			p-value Un-adjusted			Adjusted OR			p-value Adjusted		
Symptoms	Smear n = 9,120	Xpert n = 8,954	Culture n = 7,597	Smear	Xpert	Culture	smear	Xpert	Culture	Smear	Xpert	Culture	Smear	Xpert	Culture
Coughing	91	149	110	3.40	1.45	1.95	< 0.0001	0.0041	< 0.0001	2.56	1.42	2.13	0.0009	0.0559	0.0005
Cough > 2 weeks	82	124	94	2.69	1.31	1.73	< 0.0001	0.0359	0.0005						
Cough < 2weeks	13	25	16	1.45	1.37	1.38	0.2072	0.1380	0.2239						
Cough with sputum	68	99	70	2.58	1.35	1.50	< 0.0001	0.0207	0.0109	1.30	0.97	0.89	0.2774	0.8883	0.5931
Hemoptysis(Blood Cough)	9	17	9	1.86	1.73	1.39	0.0713	0.0316	0.3460	0.72	1.20	0.91	0.3871	0.5076	0.8087
Weight loss	30	36	28	4.01	2.08	2.45	< 0.0001	0.0001	< 0.0001	3.21	1.89	2.14	< .0001	0.0014	0.0010
Drenching night sweats	41	71	45	1.34	1.05	1.01	0.1269	0.7122	0.9512	0.90	1.02	0.81	0.6405	0.9104	0.3031
Fatigue	45	62	48	1.64	0.92	1.17	0.0084	0.5922	0.3660	1.21	0.80	1.05	0.3926	0.1962	0.8235
Fever	33	54	41	1.63	1.23	1.48	0.0162	0.1853	0.0319	1.12	1.21	1.35	0.6190	0.2897	0.1458
Shortness of Breath	24	43	22	1.92	1.63	1.23	0.0038	0.0040	0.3737	1.44	1.58	1.09	0.1371	0.0128	0.7214
Chest pain	68	105	73	1.80	1.03	1.17	0.0012	0.7944	0.3242	1.12	0.86	0.89	0.5980	0.3052	0.5036
Other symptoms	4	7	8	0.68	0.58	1.01	0.4424	0.1543	0.9851	0.56	0.56	0.94	0.2756	0.1471	0.8757
Total symptomatic	107	187	130	2.50	1.06	1.32	0.0006	0.6785	0.1513	2.73	1.13	1.41	0.0003	0.4271	0.0799

Of 305 survey cases, 6 cases were identified with rifampicin resistance; 2 from both culture and Xpert MTB/RIF tests; and 4 by Xpert MTB/RIF alone. Among the 4 diagnosed by Xpert MTB/RIF test alone, one was rifampicin sensitive while the other 3 had as no bacillary growth by culture(S1 and S2 excel).

The HIV prevalence among the 245 survey cases with known HIV status was 16.7% (n = 41). There were no differences in symptom presentation between the HIV positive and HIV negative survey cases, both before and after adjusting for the socio-demographic and clinical characteristics (Table 10).

**Table 10:** Association between TB related symptoms and HIV status among the 245 survey cases with known HIV status.

Symptoms	Number of HIV positive cases	Symptomatic (% cases)	Asymptomatic (% cases)	Un-Adj OR	Un-Adj 95%CI(OR)	p-value	Adj OR	Adjusted 95%CI(OR)	p-value
Coughing	24	147 (16%)	98 (17%)	0.93	(0.47, 1.84)	0.8340	0.63	(0.21, 1.95)	0.4248
Cough > 2 weeks	20	123 (16%)	122 (17%)	0.93	(0.48, 1.83)	0.8416			
Cough < 2weeks	4	24 (17%)	221 (17%)	0.99	(0.32, 3.08)	0.9925			
Cough with sputum	16	94 (17%)	151 (17%)	1.03	(0.52, 2.06)	0.9245	1.59	(0.54, 4.7)	0.4041
Blood Cough	2	15 (13%)	230 (17%)	0.75	(0.16, 3.47)	0.7157	0.52	(0.09, 3.01)	0.4621
Weight loss	7	36 (19%)	209 (16%)	1.24	(0.5, 3.07)	0.6372	1.12	(0.33, 3.83)	0.8569
Drenching night sweats	9	71 (13%)	174 (18%)	0.64	(0.29, 1.43)	0.2770	0.42	(0.15, 1.22)	0.1101
Fatigue	13	65 (20%)	180 (16%)	1.36	(0.65, 2.81)	0.4106	1.19	(0.45, 3.14)	0.7272
Fever	13	53 (25%)	192 (15%)	1.90	(0.91, 4)	0.0860	2.54	(0.92, 7)	0.0723
Shortness of Breath	8	40 (20%)	205 (16%)	1.30	(0.55, 3.08)	0.5453	1.00	(0.35, 2.88)	0.9971
Chest pain	21	108 (19%)	137 (15%)	1.41	(0.72, 2.77)	0.3131	1.76	(0.75, 4.12)	0.1958
Other symptoms	1	11 (9%)	234 (17%)	0.49	(0.06, 3.9)	0.4871	0.48	(0.05, 4.69)	0.5311
**Total symptomatic**	**33**	**186 (18%)**	**59 (14%)**	**1.38**	**(0.6, 3.17)**	**0.4533**	**1.72**	**(0.68, 4.35)**	**0.2534**

Among the survey prevalent cases with any TB related symptoms n = 225, 146 (64.9%) had not sought any health care for their symptoms prior to the survey. Of the 75 symptomatic TB cases that sought care, 58 (77.3%) went to county public hospitals while 16 (21%) visited pharmacies and private practitioners. Among the survey prevalent cases who presented with symptoms and did not seek health care, 75% reported that they thought their symptoms were not serious enough to warrant medical attention(S1 and S2 excel).

Among the survey participants with at least one symptom, 3,948 (16%) reported that they had sought health care for the symptoms and 19,463 (80%) reported not seeking for care at all, while 845 (3.5%) gave no response. Of those that did not seek health care, 43.7% of them gave no reason while 10,957 (56.3%) had varying reasons of which a majority (82%) felt that the symptoms were not serious to warrant care.

## Discussion

The prevalence of bacteriologically confirmed pulmonary TB in those above 15 years in Kenya was found to be 558 (455–662) per 100,000 population. It is comparable with findings in Uganda 401(95%CI: 292–509) per 100,000 adult population, Nigeria 524 (378–670) per 100,000 adult population and Zambia 638 (502–774) per 100,000 adult population but higher than that reported in Tanzania 295 per 100,000 adult population and Ethiopia 277 (208–347) per100, 000 population [10–15].

WHO extrapolation of the survey prevalence to all forms of TB and all ages results in an overall prevalence of 426 (347–504) per 100,000 population in 2016 [10]. Compared to the 2016 reported notification rate for Kenya, the prevalence to notification ratio is 2.5:1 [10]. It also results in an upward revision of the TB incidence rate to 348 (213–516) per 100,000 in 2016, compared to the WHO pre-survey estimate of 233 per 100,000 (95% CI 188–266) per 100,000 in 2015 [1].

Using the current incidence, about 169,000 (103,000–250,000) people fell ill with TB disease in 2016, but only 46% (77,376) were diagnosed and put on treatment [1]. Kenya is thus facing a high burden of TB and 54% of the people estimated to have TB remain un-notified.

Gender disparity in health seeking behaviour has been observed in HIV and TB care showing a greater reluctance among men to seek health care when sick [16–18]. In the survey confirmed cases, majority (65%) of those with symptoms who did not seek treatment were men. This, together with the finding that men had a disproportionately high burden of TB- two and half times that observed in females and twice more than that reported through routine surveillance shows that Kenya needs to develop specific approaches to remove any access barriers, reduce delays in diagnosis and improve management of TB among men.

There was a prevalence peak at 25–34 year age-group and the young age groups (15–44 years) contributed to two thirds of the survey prevalent cases, suggesting that TB disease in Kenya is marked by active transmission in the community. This is unlike Tanzania’s findings where majority of the cases were 45 years and older, indicative of progression from earlier latent infection [14].

In addition, the prevalence to notification gap was highest in the age group 25–34 and those over 65-year-old. This indicates that there are many cases in this age groups who are not notified or not diagnosed. In the inventory study, under-reporting was found to be higher in those over 55 years old which correlates with what was found in this survey [19]. Operational research should therefore be carried out to identify risk factors and understand why TB is being missed in this two age groups.

Majority (83%) of the prevalent TB cases were HIV-negative, suggesting that a large burden of TB also exists in the HIV un-infected population and highlighting the need to equally intensify TB case finding strategies in this population. The lower TB/HIV co-infection rate among the prevalent TB cases (16.7%) compared to notified TB cases (31%) [3] was also observed in in Uganda [15] and has been largely attributed to the effective implementation of HIV interventions such as increased antiretroviral therapy coverage, a situation likely to further improve with current implementation of test and treat approach and the use of more efficacious ART regimens. In addition, people living with HIV already enrolled in care, unlike their HIV-negative counterparts, have a higher likelihood of early TB diagnosis due to regular screening encountered during their scheduled routine clinic visits.

However, we caution that against losing focus on further accelerating the implementation of TB/HIV interventions as the low co-infection rate could also be explained by the high mortality associated with undiagnosed TB among people living with HIV in the community that could have concealed the actual burden of TB/HIV co-infection as found by this survey. In addition, of the estimated 53,000 (32,000–79,000) incident TB/HIV co-infected cases in Kenya in 2016, only 43% of them were notified, implying existence of a similarly large case detection gap for TB among people living with HIV [10].

The survey shows a higher burden of TB in urban (760 per 100,000 population) compared to rural settings (453 per 100,000 population) consistent with routine TB data which shows higher notification in the big cities [20]. These findings highlight need to focus on urban TB care and prevention to address this skewed burden of TB among the urban population, 60% of whom live in low income informal settlements [21]. Focus on ending urban TB should include addressing the influence of the associated broader social and economic factors [10]. These broader influences include level of food poverty with a prevalence of 32% in 2016, implying that 14.5 million individuals were at risk of malnutrition, level of poverty, with 36.1% of people living below the national poverty line in 2016; and low coverage of health insurance at 19%, leading to financial barriers to accessing health services[22,23]. It would require the Ministry of Health to go beyond the current purely health-led response and coordinate across a range of government departments addressing health, education, social protection, housing, agriculture and poverty [24].

Based on this prevalence survey, the percentage of individuals with TB symptoms who had not sought care was 67%. They may not have been experiencing severe symptoms yet, or they faced barriers to care that made them not to make it to the health system. The TB patient costs study found that TB can impose profound costs on families reporting that a third of TB affected households and two thirds of drug-resistant TB affected households experience catastrophic costs as they seek care for TB [23]. In addition, it highlighted that TB is a cause of poverty, with 28% of TB patients using negative coping mechanisms like taking a loan, use of savings and sale of assets to meet the expenses for seeking TB diagnosis and care. This means that people may not access healthcare due to financial difficulties and addressing these financial barriers may encourage more individuals in the community to seek care for TB and help close the current case detection gap.

In this survey, 80% of the symptomatic participants and 67% of confirmed TB cases with at least one TB related symptom did not seek health care because they did not perceive the symptom as being serious. Perceived severity of illness and the number of symptoms has been noted to be a predictor of health seeking behavior[18]. These findings call for sufficient investment in community TB health communication to increase awareness and encourage people to seek early intervention for symptoms [25]. Health interventions in Reproductive Maternal Neonatal and Child Health in parts of Kenya have successfully invested in this approach by using school health programs to target children as change agents for reaching their families with health messages[26]. In addition, strengthening systematic screening of selected high-risk groups like all contacts of people with TB can help identify patients with early symptoms.

Data from this survey and the TB inventory survey [19] shows that they are people who actively seek health care but for whom the systems fail to diagnose and report their care status. In this survey, 80% of those who sought care with TB symptoms, did not get diagnosed at initial contact with the health facility for various reasons. Possible solutions lie in optimizing the TB care cascade to eliminate leakages for persons who have accessed care at all levels of the health care system and developing and implementing approaches to systematically screen all persons seeking care in all health facilities for TB.

According to the TB patient pathway analysis, only 43% of TB patients’ encountered diagnostic capacity at the health facility they initiated care [27]. This misalignment between availability of diagnosis and care seeking often delay diagnosis and discourage completion of TB diagnostic cycle [27]. As recommended by the study, strengthening specimen referral systems could help close the health system related detection gaps and accelerate access to diagnosis. In addition, the TB inventory survey and a recent TB epidemiological review showed that one fifth of bacteriologically confirmed TB cases in Kenya are not notified to the NTP due to initial loss-to follow from the laboratory [19,28]. These patients though diagnosed were not linked to TB treatment. There should therefore be deliberate mechanisms to link laboratory and clinical information to minimize pre-treatment loss to follow up. This can be achieved by implementing a system which pulls data from laboratory systems into the TB information system as well as a more robust follow up of all laboratory registered bacteriologically confirmed cases and patients diagnosed in-patient settings could help to close this gap [28].

This survey found that 21% of the respondents first sought health services from private practitioners and private pharmacists. These findings are reinforced by the PPA findings that 27% of presumptive TB patients sought services in the formal private sector and the informal sector, including pharmacies, was the initial point of care for another 15% [27]. Several supporting partners have been playing a catalytic role in sustaining the public private partnership approach in the country [29]. Under this approach, the engaged facilities use national policies and guidelines, recording and reporting tools, and government provides drugs and other essential commodities for treatment. Engaged private facilities benefit from routine supervisory visits by the National TB Program. However, there has been limited engagement beyond established hospitals and clinicians in urban areas [30]. The private sector contributes to 18% of the total TB patient notification against 42% of patients with presumed TB seeking care there [27]. In addition, unreported TB cases in Kenya are more likely diagnosed at private health facilities [19]. This gap in actual notification and care seeking highlights an important opportunity for a more comprehensive TB public private engagement approach in Kenya. To address this the NTP should expand the coverage of TB services to this sector while enhancing the involvement of all private practitioners including pharmacies and informal health providers with a specific task mix for each provider category in order to find the missing cases.

The combination of cardinal symptoms of cough of more than two weeks, fever, night sweats and weight loss would have missed 41% of the prevalent cases. Testing all people with any symptom consistent with TB—cough of any duration, hemoptysis, night sweats, weight loss, fatigue, fever, and shortness of breath—would have substantially increased the case yield to 74%. These survey findings highlight the need to revise the symptoms duration and combination used to define a presumptive TB patient in Kenya as the full case-finding potential of chest x-ray and Xpert will not be realized if there is a restrictive symptom requirement up-front [31]. In 26% of the survey cases, active TB disease seemed to have preceded the development of symptoms as they did not have any of the symptoms of cough, fever, weight loss, night sweats, fatigue, breath shortness nor chest pains.

Chest x-ray had a much higher yield detecting 88% of the prevalent cases. Over 50% of the confirmed TB cases had no classical TB symptoms but had an abnormal chest X-ray. Chest X-ray screening alone helped to identify an additional 42% of the prevalent cases; similar to the findings in Zambia (39%) [11]. Considering that Kenya uses symptom screening for identification of those with presumptive TB, this survey shows that an approach that excludes chest X-ray screening misses a large proportion of TB cases and calls for its urgent local adaptation.

While X-ray may be very useful in diagnosing symptomatic paucibacillary TB in a clinic setting, the added value in asymptomatic individuals in the population may be relatively minor. In this survey, the prevalence of TB in asymptomatic individuals was approximately 2 per 1,000, suggesting that screening asymptomatic individuals for TB with chest x-ray would be a relatively low yield activity.

One key feature of this survey is that it employed smear microscopy, culture and Xpert MTB/RIF for diagnosis. Of the survey cases identified, 60% were smear negative, meaning that these cases could have been missed by routine case detection that relies on microscopy only. The use of Xpert MTB/RIF identified an additional 29.5% prevalent cases. The low performance of culture compared to Xpert MTB/RIF could be attributed to reduced viability of the bacilli during sputum sample transport to the National TB Reference Laboratory. The survey therefore recommends replacement of smear microscopy with a rapid point-of-care diagnostic test, such as Xpert MTB/RIF across all health facilities. Considering that Xpert MTB/RIF equipment has been placed in only 188 health facilities in 2018, the current sputum specimen transportation system, which has been found to be weak and inadequate in terms of coverage and frequency must be strengthened to increase testing coverage [30].

In conclusion, we found that the prevalence of TB in Kenya is higher than had been previously estimated, and about half of the people who fall ill with the disease are missed each year.

## Supporting information

S1 Excel Information line data for the Kenya TB prevalence survey 2016. (CSV)

S2 Excel Data dictionary for the information line data for the Kenya TB prevalence survey 2016. (XLS)
