# Correction: Kenya tuberculosis prevalence survey 2016: Challenges and opportunities of ending TB in Kenya

**DOI:** 10.1371/journal.pone.0211593

**Published:** 2019-01-25

**Authors:** The PLOS ONE Staff

The affiliation for the last author is incorrect. Herman Weyenga is not affiliated with #8 but with #1 Ministry of Health, Nairobi, Republic of Kenya. The publisher apologizes for this error.
